# Effect of Fishing Season and Size on the Physicochemical and Microbiological Characteristics of Salted Sardines (*Sardina pilchardus*)

**DOI:** 10.1155/2024/9376432

**Published:** 2024-02-19

**Authors:** Charifa Aoua, Bouchra Yacoubi, Abderrahmane Zekhnini

**Affiliations:** Department of Biology, Aquatic Systems Laboratory, Marine and Continental Environments, Faculty of Science, 8106 Agadir, Morocco

## Abstract

Salting is the preferred method used by manufacturers to preserve the quality of fish. The aim of this study was to investigate the effect of size and fishing season on the physicochemical and microbiological characteristics of sardines. Two batches of sardines, caught, in winter and summer, were sorted according to size, salted, matured, and stored at 18°C. A control batch, consisting of sardines from the summer batch, was also prepared without subcutaneous fat. Various physicochemical and microbiological parameters were monitored during 12 weeks of maturation. The recorded values showed a decrease in pH, moisture content, and water activity (a measure of the water available for biological and chemical processes), while salt concentration increased. When comparing samples, the lowest values for histamine (94.3 and 48.3 mg/kg) and lipids (9.3 and 8.2%) were observed in the large and small sardines of the winter batch, respectively. In the summer batch, higher values were recorded for histamine (847.3 and 127.9 mg/kg) and lipids (14.5 and 8.6%) in the large and small sardines. Furthermore, the removal of fat in control of the summer batch resulted in lower histamine accumulation compared to the batch with fat. The microbiological parameters showed a decrease in the number of nonhalophile bacteria, while the number of halophile bacteria increased. This study showed a strong correlation between three important factors, fishing season, fish size, and histamine content, which can contribute to the successful salting of sardines and ensure the wholesomeness of the final product.

## 1. Introduction

Morocco has emerged as a global leader in sardine production and exports, owing to its unique geographical advantage of a dual coastline along the Atlantic Ocean and Mediterranean Sea. This success can be attributed to its strategic approach, emphasizing quality control throughout the production chain, as highlighted by the FAO in 2016 [1].

Beyond the substantial economic impact, sardines offer numerous health benefits for consumers. Its regular consumption has been shown to help prevent various diseases, including diabetes, cardiovascular ailments, and inflammatory cancers [2]. Ensuring proper preservation of this fish is essential, as it safeguards healthful attributes by minimizing the loss of vital nutrients like amino acids, trace elements, fat-soluble vitamins, and polyunsaturated fatty acids [3]. However, the quality of fish can be influenced by various factors, such as seasonal environmental fluctuations in growth, sex, age, and size of the fish, as well as the fishing area, salinity, and seawater temperature [4, 5].

One of the most common methods for preserving fish is the use of salt. This involves treatment with precise amounts of salt and enzymatic maturation over a span of two to eight months. Enzymatic maturation is a complex sequence of reactions depending on physicochemical conditions (temperature, salt content, water activity, and pH) and fish biology (lipids, enzymes, bacteria, etc.) [6]. Several factors contribute to the maturation of sardines, with the inherent qualities of fish and salt playing a significant role. Other factors include temperature, pressing time, and maturation duration [7]. Additionally, the salting technique causes partial dehydration of the fish, which inhibits enzymatic and microbial activity, giving the product a distinctive taste and consistency [8, 9].

Despite maintaining its global leadership position, the salted fish industry faces challenges that impact product quality. When salt fails to penetrate fish tissues or remove water effectively, it can encourage microbial growth, leading to elevated histamine levels and posing a health risk to consumers. Consequently, our study is aimed at mastering the art of salting to ensure a healthy, high-quality product. To achieve this, we assessed the impact of sardine size and fishing season on various physicochemical parameters, including lipids, pH, and salt concentration, as well as health-related factors such as histamine levels and microorganism content in sardine tissues.

## 2. Materials and Methods

### 2.1. Sampling Conditions

This work was carried out on 100 kg of excellent-quality sardines (*Sardina pilchardus*) caught in the Atlantic Ocean in 2019 (south of Tantan, Morocco) in two seasons and two batches: batch A (January-March) and batch B (July-September). The choice of these sampling seasons was based on the fattening and emaciation phases of the sardine [10].

Each batch was divided into two groups: large size (21.5 cm) and high weight (83 g) and small size (12 cm) and low weight (27 g). Ten kilograms of batch B sardines, from which the skin and subcutaneous fat have been removed, was used to study salt penetration speed.

### 2.2. Salting Conditions

After each batch was received at the laboratory, the sardines were kept on ice (0-1°C), washed, scaled, and gutted by hand, followed by 35% NaCl dry salting. The salting used fine-grained iodized salt. After salting, the sardines were divided into 10 kg plastic buckets and then pressed using a press (NAVAPRO, Spain) at 90 g/cm^3^ during the first three weeks of ripening and at 40 g/cm^3^ during the remainder of the ripening phase. All batches were stored at 18°C for 12 weeks of ripening [11].

### 2.3. Chemical Analysis

Daily samples of sardines were taken for the analysis of flesh salt content, and weekly samples were taken for the following chemical analyses. After homogenizing 10 g of fish sample in 100 ml of distilled water, the pH of the muscle was measured (Hanna Instruments, Italy) fitted with an electrode. Moisture was determined using the AOAC technique [12]. Water activity (Aw) was determined by the HygroPalm 23-AW-A (Rotronic AG, Switzerland) at 27°C. Chlorides were determined using the AOAC method [13]. Lipids were extracted using the Soxhlet method AOAC [14]. Histamine determination was performed using a fluorimeter (Turner Designs Trilogy 10-AU-904, USA) according to the method of Lerke and Bell [15].

### 2.4. Microbiological Analysis

Three microbial groups were enumerated: psychrotrophic and mesophilic flora from the receipt of fresh sardines to the end of the experiment and halophilic flora from the 4th week of ripening to the end of the experiment. The culture medium used in this work was plate count agar (PCA) (Condalab, Spain). Psychrotrophic bacteria were incubated at 6°C for 10 days and mesophilic bacteria at 30°C for 72 hours. Halophilic bacteria were counted on a PCA medium containing 20% NaCl and incubated at 37°C for 48 hours [16].

### 2.5. Statistical Analysis

The results are reported as mean values ± standard deviation. All experiments were performed in triplicate (*n* = 3). The level of significance of the difference (*p* < 0.05) was determined by Fisher's ANOVA test (STATISTICA 6 software).

## 3. Results and Discussion

### 3.1. Variation in pH, Water Activity, Moisture, and Lipids during Ripening

**Figure 1:**
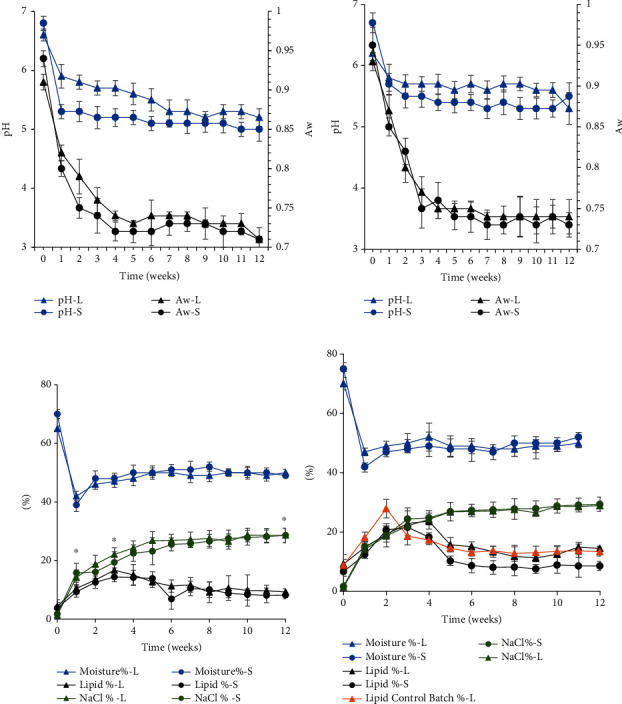
Physicochemical changes in salted sardine: moisture values, lipid content, NaCl, pH, and water activity (Aw) observed sardines from (a, c) batch A and (b, d) batch B. L: large; S: small. Data were expressed as mean ± standard deviation (*n* = 3). Values (∗) indicate a significant difference between batches A and B of large sardine samples (*p* < 0.05).

On the first day of ripening, the sardine muscle pH decreased from 6.8 ± 0.1 to 5.1 ± 0.2 in batch A and from 6.7 ± 0.2 to 5.5 ± 0.3 in batch B, irrespective of size (Figure 1). These results are in line with reports by Tsai et al. [17] and Kuda et al. [18] on salted and fermented fish. The decrease in pH was often attributed to the release of lactic acid and free fatty acids.

The decrease in pH was accompanied by a drop in water activity to below 0.73 until the end of the experiment (Figure 1). With the same variation, water diffusion was more rapid and significant at the end of the first week of ripening. From an initial moisture content of 65 ± 1.5% and 70 ± 2.9%, this rate reached 42 ± 2.3% and 39 ± 1.6%, respectively, in large and small sardines from batch A and from 70 ± 2.1% and 75 ± 0.9% to 47 ± 1.3% and 42 ± 1.8%, respectively, in large and small sardines from batch B. After 3 weeks of ripening, the moisture content increased and stabilized at values between 49 ± 1.2% and 51 ± 0.8% in all batches (Figure 1). Our results are in accordance with those reported by Mohhdaly et al. [19] on salted sardines matured at a temperature of 22 ± 2°C. The drop in moisture was caused by the salt content (osmosis effect) and the high pressure applied, which caused the exudation of some of the salty water present in the muscle tissue. Reduced moisture in the food and lower water activity extend shelf life and prevent the spread of pathogenic microorganisms [20].

According to Figure 1, lipid contents show an inverse evolution to that of flesh moisture. At reception, sardines of batch A had an average lipid content of 4.4 ± 0.9% for large sardines and 3.8 ± 1.9% for small ones, while batch B large sardines had a lipid content of 9.2 ± 0.7% and 6.7 ± 1.6% for small ones. After 4 weeks of ripening, lipid levels significantly increased in both large and small sardines, reaching 15.1 ± 3.6% and 14.2 ± 2.5% in batch A and 23.7 ± 2.7% and 18.3 ± 3.4% in batch B, respectively. Several authors associated the evolution of lipid levels during maturation with the initial lipid content and water loss caused by pressure on the sardine [21, 22]. Our data suggest the superiority of summer-salted sardines in terms of lipid content during ripening over winter-salted sardines. The increase in lipid content confirms successful salting [23]. A decrease in lipid levels was recorded from week 6 onwards in large and small sardines, respectively, 11.3 ± 2.1% and 6.9 ± 3.5% in batch A and 15.1 ± 1.6% and 8.1 ± 2.4% in batch B. The decrease in lipid levels at the end of the ripening period could be due to their insolubility in water and their diffusion through the cell walls, the hydrolysis of phospholipids and triglycerides, or the release of free fatty acids that were soluble in water [24, 25].

### 3.2. Evolution of Salt Content during Ripening

Figures 1(c) and 1(d) show the evolution of salt content as a function of time in the flesh of sardines from both batches A and B. After one day of salting, the salt content increased in batch A, reaching 15.4 ± 0.3% and 16.2 ± 0.6% in large and small sardines, respectively. Similarly, the salt levels in sardines from batch B were 14.2 ± 0.6% and 15.5 ± 2.1% in large and small sardines, respectively. Monitoring confirmed rapid salt penetration simultaneously in both batches. The rate at which sodium chloride penetrates the fish depends, in particular, on the difference between the salt concentration in the flesh and that in the medium, the body size, and the fattening state of the fish [26, 27]. In the present study, after one day of salting, the salt content increased significantly, reaching levels above 15% for all sardine batches. This value corresponds to that recommended by CODEX [28]. No significant differences (*p* < 0.05) were observed between the small sample sizes of batches A and B. They showed a similar evolution of salt penetration, even if the sardine fishing period was different. However, the larger sardines showed a significant difference between (*p* < 0.05) batches A and B. A slight slowdown in salt penetration was observed in the large sardines of batch B. This may be explained by the change in the muscle structure of the fish, which affects the salt retention capacity and the amount of subcutaneous fat in the fish, particularly those caught in the summer, which corresponds to the fattening phase.

### 3.3. Histamine Contents

**Figure 2:**
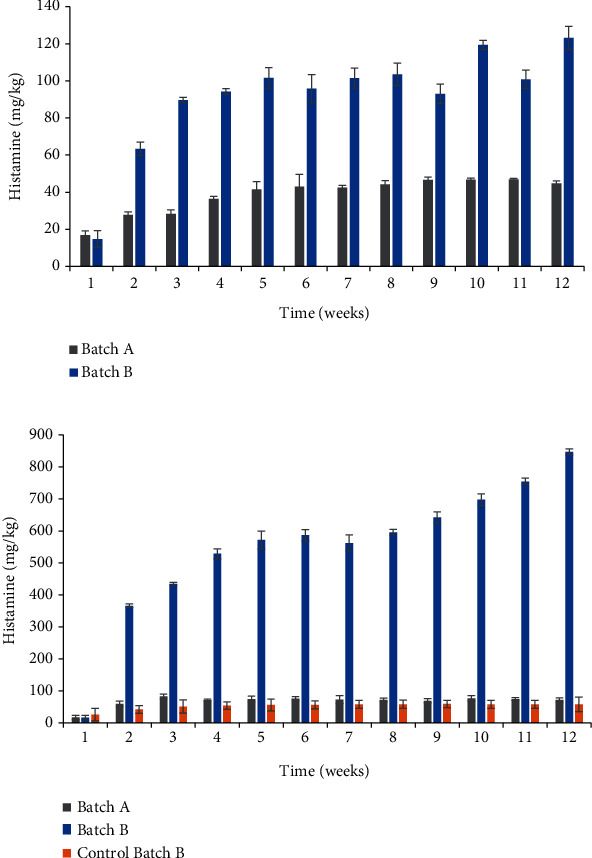
Histamine levels in (a) small and (b) large salted sardines from batches A and B during 12 weeks of ripening. Data were expressed as mean ± standard deviation (*n* = 3).

Initially, the average histamine levels in fresh sardine flesh ranged from 14.7 ± 4.5 to 17.9 ± 2.1 mg/kg. These levels varied considerably throughout the ripening process. During 12 weeks of ripening, histamine levels in large sardines from batch A increased significantly, reaching 94.3 ± 6.2 mg/kg for sardines. However, histamine value in batch B for sardines of the same size is 847.3 ± 9.6 mg/kg (Figure 2(b)). The values reached were above the permitted level for semipreserved products [29]. Histidine decarboxylation significantly increased in summer sardines, particularly in large sardines as reported by Osako et al. [30].

On the other hand, the lowest histamine values were recorded in samples of small sardines from batch A (48.3 ± 1.4 mg/kg). Moreover, histamine levels in small samples from batch B (127.9 ± 2.1 mg/kg) were three times higher than those in batch A (Figure 2(a)). However, this level corresponds to the authorized limit by food safety authorities [29, 31].

Histidine decarboxylation was slowed down at the third week of maturation in small fish and continued to evolve in large sardines. The difference in histamine values as a function of the fishing season observed in this study is similar to the findings of a survey on the Japanese sardine *Sardinops melanostictus* [32]. These authors reported that the highest histidine content was noted in July, August, and September and the lowest in February and March. The sudden increase in histamine may be attributed to the significant decarboxylation of histidine in sardines caught in the summer [33, 34].

### 3.4. Effect of Sardine Fat Removal on Physicochemical Characteristics

The amount of subcutaneous fat removed was 26 ± 2 g, 22 ± 4 g, and 28 ± 1 g, respectively, for the months of July, August, and September (results not shown). The results obtained for defatted fish from batch B show similar results for the physicochemical parameters for the same sardines without defatting (Figure 1). The expulsion of liquids and the admission of salt into the flesh are not always influenced in the same proportion by the presence of a barrier made up of fat and skin. At the end of the ripening process, there was a significant increase in lipid values from 8.8 ± 3.7% to 12.8 ± 2.7% (Figure 1(d)) and in histamine from 26 ± 1.9 to 58 ± 2.1 mg/kg (Figure 2(b)). On the other hand, histamine levels were significantly higher (847.3 ± 9.6 mg/kg) in sardines from which the fat had not been removed than in defatted sardines (58 ± 2.1 mg/kg) (Figure 2(b)). This result indicates that the amount of subcutaneous fat could be a disadvantage for the salting preservation technique.

### 3.5. Microbiological Study

Many factors influence fish flora, with temperature, water activity, pH, and salt content being the most important [35, 36].  Table 1 shows that the number of psychrotrophic and mesophilic bacteria did not show any significant difference (*p* < 0.05) between sardines with and without subcutaneous fat. As maturation progressed over 12 weeks, there was a significant reduction in the number of mesophilic and psychrotrophic bacteria. In batch A, the number of mesophilic bacteria decreased from 7.9 ± 0.2 to 3.6 ± 0.3 log cfu/g, and the number of psychrotrophic bacteria decreased from 5.5 ± 0.1 to 3.4 ± 0.2 log cfu/g. In batch B, the number of bacteria increased from 8.3 ± 0.2 to 4.0 ± 0.1 log cfu/g for mesophilic bacteria and from 5.4 ± 0.1 to 3.6 ± 0.3 log cfu/g for psychrotrophic bacteria. Our findings align with those reported by Karaçam et al. [16] on anchovies salted with a concentration of 26% salt and stored at room temperature for 12 weeks. The decrease in mesophilic and psychrophilic flora was associated with the initial load, high salt concentration, and a decrease in fish pH [37, 38]. Except for halophilic bacteria, a significant increase (*p* < 0.05) in the number of bacteria was observed in defatted batch B sardines, reaching 9.9 ± 0.2 log cfu/g instead of 6.5 ± 0.1 and 7.2 ± 0.2 log cfu/g, respectively, in defatted batch A and batch B sardines. Various studies focused on halophilic bacteria, which were commonly found in salted fish [39, 40]. Salt plays a crucial role in inhibiting the growth of pathogenic bacteria. Histamine-producing bacteria were mostly inactive at high salinity, acidic pH, and refrigerated temperatures [41–43]. The authors reported that bacterial growth and histamine production were inhibited at salt concentrations of 10% to 20% [44]. However, a study conducted by Kim et al. [45] on salted anchovies, which were removed from the market due to high histamine levels, revealed that the dominant halophilic bacteria in semicanned salted anchovies belong to the *Bacillus* spp. and *Staphylococcus* spp. groups. These bacteria can produce histamine even at salt levels above 20% [46]. However, other authors reported that these bacteria produced negligible amounts of histamine in the culture medium, indicating that they were not responsible for histamine accumulation in semicanned anchovies [45]. Our findings align with this author. The halophilic microbial load was higher in defatted sardines (9.9 ± 0.2 log cfu/g) than in sardines with fat (7.2 ± 0.2 log cfu/g). Additionally, histamine levels detected in defatted sardines were lower (58 ± 2.1 mg/kg) compared to sardines with fat (847.3 ± 9.6 mg/kg). These results lead to the conclusion that the level of histamine produced in fish does not appear to depend solely on the number of bacteria but also on other crucial factors such as lipid content. Consequently, obtaining a final product with the desired organoleptic and physicochemical characteristics can be achieved before 12 weeks of maturation.

The results obtained in this work could assist salting industry professionals in mastering the salting process of sardines, addressing health issues such as high histamine levels, as well as economic challenges like the loss of raw material (sardines) and the increase in production costs.

## 4. Conclusion

This study showed that fishing season and fish's size appear to be the key elements in the processing of salted sardines. The use of salting at a high concentration and an optimally controlled temperature cannot curb histamine formation in large lipid-rich sardines. The results exhibited a good correlation between the evolution of histamine in sardines during maturation, the fish's fat content, and the species' size, making a significant contribution to global food security by providing healthy products and generating significant economic impact.

## Figures and Tables

**Table 1:** Evolution of bacterial content (log cfu/g).

Sample	Bacteria	Initial		4 weeks		8 weeks		12 weeks	
		Batch A	Batch B	Batch A	Batch B	Batch A	Batch B	Batch A	Batch B
With skin	Mesophilic	7.8 ± 0.2	8.3 ± 0.2	6.5 ± 0.2	6.8 ± 0.2	5.6 ± 0.2	5.5 ± 0.3	3.6 ± 0.4	4.0 ± 0.1
	Psychrotrophic	5.5 ± 0.1	5.4 ± 0.1	4.9 ± 0.3	4.9 ± 0.2	3.8 ± 0.1	4.1 ± 0.1	3.4 ± 0.2	3.6 ± 0.3
	Halophilic	—	—	5.3 ± 0.2	4.5 ± 0.5	5.7 ± 0.2	5.7 ± 0.2	6.5 ± 0.1	7.2 ± 0.2^B^

Without skin	Mesophilic	—	8.3 ± 1.2	—	5.5 ± 0.3	—	3.6 ± 0.3	—	2.2 ± 0.2
	Psychrotrophic	—	5.5 ± 0.1	—	4.4 ± 0.2	—	3.3 ± 0.1	—	1.6 ± 0.1^E^
	Halophilic	—	—	—	6.3 ± 0.3^A^	—	7.6 ± 1.2^C^	—	9.9 ± 0.2^D^

Data are expressed as mean ± standard deviation (*n* = 3). Values with different letters are significantly different (*p* < 0.05) between samples with and without skin.

## Data Availability

The data that support the findings of this study are available on request from the corresponding author.
